# Effects of a community-based data for decision-making intervention on maternal and newborn health care practices in Ethiopia: a dose-response study

**DOI:** 10.1186/s12884-018-1976-x

**Published:** 2018-09-24

**Authors:** Ali Mehryar Karim, Nebreed Fesseha Zemichael, Tesfaye Shigute, Dessalew Emaway Altaye, Selamawit Dagnew, Firew Solomon, Mulu Hailu, Gizachew Tadele, Bantalem Yihun, Nebiyu Getachew, Wuleta Betemariam

**Affiliations:** 1 The Last Ten Kilometers Project (L10K) 2020, JSI Research & Training Institute, Inc, Bole Sub-City, Kebele 03/05, Hs # 2111, Addis Ababa, Ethiopia; 2 Ethiopia Performance Monitoring and Evaluation Service (EPMES), Social Impact, Bole Sub-City, Woreda 13, House # 478, Addis Ababa, Ethiopia

**Keywords:** Community-based information system, Community engagement, Community health workers, Women’s development army of Ethiopia, Health extension program of Ethiopia

## Abstract

**Background:**

Community participation and community health volunteer programs are an essential part of the health system so that health services are responsive and accountable to community needs. Information systems are necessary for community health volunteer programs to be effective, yet effectiveness evaluations of such information systems implemented at scale are rare. In October 2010, a network of female volunteers with little or no literacy, the Women’s Development Army (WDA), was added to extend Ethiopia’s Health Extension Program services to every household in the community. Between July 2013 and January 2015, a health management information system for the WDA’s Community-Based Data for Decision-Making (CBDDM) strategy was implemented in 115 rural districts to improve the demand for and utilization of maternal and newborn health services. Using the CBDDM strategy, Health Extension Workers (HEWs) fostered the WDA and community leaders to inform, lead, own, plan, and monitor the maternal and newborn health interventions in their *kebeles* (communities). This paper examines the effectiveness of the CBDDM strategy.

**Methods:**

Using data from cross-sectional surveys in 2010–11 and 2014–15 from 177 kebeles, we estimated self-reported maternal and newborn care practices from women with children aged 0 to 11 months (2124 at baseline and 2113 at follow-up), and a CBDDM implementation strength score in each kebele. Using kebele-level random-effects models, we assessed dose-response relationships between changes over time in implementation strength score and changes in maternal and newborn care practices between the two surveys.

**Results:**

Kebeles with relatively high increases in CBDDM implementation strength score had larger improvements in the coverage of neonatal tetanus-protected childbirths, institutional deliveries, clean cord care for newborns, thermal care for newborns, and immediate initiation of breastfeeding. However, there was no evidence of any effect of the intervention on postnatal care within 2 days of childbirth.

**Conclusions:**

This study shows the extent to which an information system for community health volunteers with low literacy was implemented at scale, and evidence of effectiveness at scale in improving maternal and newborn health care behaviors and practices.

## Background

The World Health Organization envisions community engagement as a critical component of health care delivery systems [1–3]. Community engagement and community-based health programs are expected to ensure that health services are responsive to individual and community needs, adaptive to local cultural practices that influence health care behavior, accountable to the community, and desirable [2, 4–7]. Consequently, community engagement strategies and voluntary community health worker programs are increasingly being used to improve access and utilization of health care services in many settings [4, 5, 8]. For the community engagement and voluntary community health worker programs to achieve their objectives, community-led monitoring and evaluation of health service delivery, including a community-based health information system, are essential [9–11].

Community-led monitoring and evaluation, including community-based health information use, has many forms. Broadly, a community-based health information system is defined as community members actively collaborating with representatives from the health system to gather, analyze, interpret and use data to improve the efficiency of the delivery of health services to the community [9–13]. There are several examples of implementing health information systems for community-based programs to improve uptake of health services [8, 13–24]. These community-based information systems can be categorized into two types: (a) as a part of the health management information system of a national program, where salaried community health workers collect and use data to deliver and ensure quality of services, and the data are then aggregated and used at higher levels of the health system for program monitoring and planning purposes; and (b) a strictly community-level health information system in which health workers in the formal health system facilitate community health volunteers and community representatives to collect data and use it to plan and improve uptake of health services. For example, the Community Health Information System of the Health Extension Program of Ethiopia is part of the national health management information system [14], and the community-based health information systems of orphan and vulnerable children programs of Kenya, Tanzania, and Zambia are part of health management information systems of donor-funded programs implemented at scale [20–22]. There are several examples of strictly community-based health information systems or community-based program monitoring and evaluation systems where the information systems are implemented and used within the local community to improve the uptake of health services [18, 23–25]. Although there is evidence that the strictly community-based health information systems in Honduras, Kenya, and Nigeria were effective in improving health care utilization [13, 18, 23], and that in Uganda, community-based monitoring and evaluation improved health outcomes [24], these community-based health information systems were pilot studies, or they were implemented on a small scale.

A recent systematic literature review indicates that there is a need for more quantitative studies to assess the effectiveness of community engagement in maternal and newborn health services [8]. However, despite calls for effectiveness evaluations of large-scale programs [26], there are no examples of effectiveness in strictly community-based health information systems implemented at scale. We designed, tested and implemented Community-Based Data for Decision-Making (CBDDM) — a strictly community-based health information system for community health volunteers with little or no literacy — in 115 rural agrarian districts in the Amhara, Oromia, Southern Nations, Nationalities, and Peoples’ (SNNP), and Tigray regions of Ethiopia, covering about 17 million people, which is about 19% of the total population.

This paper is the second in a series of four, investigating innovations designed to support the flagship Health Extension Program of Ethiopia to improve the maternal and newborn health of the country. The other three papers consider the Women’s Development Army (WDA) strategy [27], the use of a Participatory Community Quality Improvement strategy [28]; and the Family Conversation strategy [29]. This study analyzes the effects of CBDDM on maternal and newborn health care practices in 83 districts covering a population of about 12 million people, a sub-set of 115 districts implementing CBDDM at the time.

## Methods

### Study settings

In Ethiopia, the rural district health system includes one primary hospital per district with 4–5 primary health care units—each primary health care unit comprises one health center with five satellite health posts to serve about 25,000 people. Health centers, staffed with health officers, nurses, midwives and laboratory technicians, provide preventive and clinical services. Health posts, each serving a *kebele* (community), are staffed by two female Health Extension Workers (HEWs) supported by a network of WDA volunteers in the community with little or no literacy, provide 18 packages of mainly promotive, preventive and selected curative services as part of the country’s flagship Health Extension Program. Since 2004, the Health Extension Program has established about 16,000 health posts and deployed about 38,000 HEWs [30–32]. The WDA is also known as the Health Development Army.

One of the initial tasks of the HEWs was to train and graduate “model households”, households that practiced proper household hygiene and sanitation and utilized basic service provisions (such as childhood vaccination and family planning) [33]. Building on the graduated model households, the Federal Ministry of Health introduced the concept of the WDA in 2010. The WDA strategy fostered community engagement to ensure that health care services were responsive to individual and community needs by engaging women volunteers from model families to disseminate health information and to facilitate uptake of high-impact interventions. Under the guidance of HEWs, the WDA network members were organized into groups, with one WDA team leader for every six WDA volunteers, each of whom was responsible for five or six households. Thus, each WDA team leader was responsible for linking 30 households in her neighborhood to use the services provided by the Health Extension Program. To date, the Health Extension Program has established a network of about 3 million WDA members [32, 34], which has led to improved maternal and newborn health care behaviors and practices [27].

### The intervention

To support the Health Extension Program to implement high-impact maternal, newborn, and child health services, the Last 10 Kilometers (L10K) Project has implemented innovative, community-based strategies (see Additional files 1–3 of the first paper in this supplement) [27]. In partnership with 12 local civil society organizations, L10K has enhanced the interactions between frontline health workers and communities to achieve more accessible, efficient, and equitable maternal, newborn, and child health services [35]. Between 2013 and 2015, the L10K Platform strategy was implemented in 115 districts, covering about 3070 kebeles*.* The platform strategy included CBDDM; Family Conversation, a forum conducted at the household of the pregnant women with her family members during the antenatal period to reinforce birth preparedness; and Birth Notification to ensure early postnatal care. The current study was limited to the 2150 kebeles from the 83 districts in which L10K implemented only its platform strategy.

**Fig. 1:**
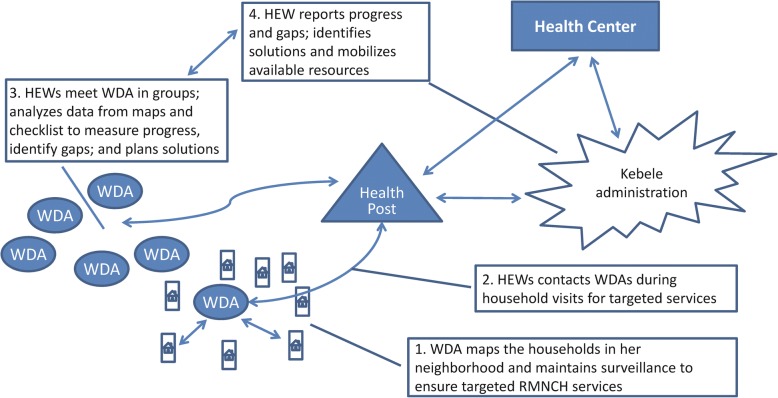
Activities involved in Community-Based Data for Decision-Making

CBDDM fostered the kebeles to generate and use data to improve maternal and newborn health practices. The strategy identified underserved households and linked them with HEWs and kebele command post leaders (kebele administrators) to address the barriers in access to maternal and newborn health services. Accordingly, HEWs were trained to support WDA team leaders to map the 30 households in their catchment areas, to keep each under surveillance, and to ensure maternal and newborn health services along the continuum of care: through pre-pregnancy, pregnancy, childbirth, postnatal care — including newborn care, to childhood immunization (Fig. 1). The surveillance system used images so that it could be maintained and updated by individuals with little or no education. HEWs collected data from WDA team leaders’ surveillance and drew on the kebele leaders to identify and address barriers to access maternal and newborn health services.

HEWs were required to visit households routinely to update the information in the Family Folders, the central piece of the Community Health Information System of the Health Extension Program [14]. Each household within a kebele has a Family Folder, which is kept at the health post. It holds information on the family members, water supply and sanitation characteristics, and maternal and child care management records [14]. The CBDDM strategy gave an effective tool to HEWs to organize their household visits and to interact with the WDA to update the Family Folders. Although the policy to implement the Community Health Information System began in 2008, the actual implementation was initiated in October 2010 [14]; thus, the Community Health Information System was being rolled out at the same time as the CBDDM strategy was beginning to be scaled-up in the L10K areas. In the L10K areas the CBDDM was an important supplement of the Community Health Information System, which helped HEWs to personify and visualize the locations of their clients, whose records they maintained in the Family Folders.

Before the CBDDM strategy was implemented, it was prototyped in selected districts, among which were 14 of the 83 districts included in this study. Nonetheless, during the study period there were no additional inputs in the prototyping areas. We anticipated that there would be natural variability in the implementation intensity of the CBDDM strategy across the 2150 kebeles in the 83 study districts.

### Study design

Using before-and-after cross-sectional household and health post surveys conducted in 2010–11 and 2014–15, we applied an internal comparison group design to examine dose-response relationships between improvements in CBDDM implementation strength in 177 kebeles during the observation period and improvements in maternal and newborn health care behaviors and practices during the same period. We measured the CBDDM implementation strength through interviews with HEWs and CBDDM activity records we obtained during the health post survey. Women with children aged 0 to 11 months in the kebeles reported care practices for their most recent pregnancy and childbirth. The hypothesis was that kebeles with a higher CBDDM implementation strength would have better improvements in maternal and newborn health care practices.

### Sample size

We used data from the before and after household and health post surveys conducted in December 2010–January 2011 and December 2014–February 2015 to evaluate the larger L10K program. The survey design for the evaluation of the broader L10K program including sample size estimation parameters for the surveys are provided in Additional files 1–3 of the first paper in this supplement [27]. For the current study, the data were first restricted to the 195 kebeles included in the surveys where only L10K Platform activities were taking place, and which were visited for data collection during both the surveys. However, data from 18 kebeles were dropped, due to missing values of some of the kebele-level variables, leaving 177 kebeles in the study. The final sample size of women with children aged 0 to 11 months was 2124 in 2010–11 and 2113 in 2014–15.

### Data collection and study participants

The household survey applied a two-stage cluster sampling method: at the first stage, kebeles were selected as primary sampling units with the probability of selection being proportionate to the population. At the second stage, the sampling strategy described by Lemeshow and Robinson (1985) was used to select the household with the target respondents [36]. The first household was selected randomly from the middle of the kebele, and from there every fifth household was visited, moving away from the middle. If the household visited had women with children aged 0 to 11 months, the women were interviewed, after seeking their consent. If a respondent was under 18 years old, then consent was sought from her husband, parents, or guardian. A quota of 12 women was interviewed from each kebele to obtain information on their socio-demographic background and the maternal and newborn health care behavior and practices associated with their most recent pregnancy and childbirth. The health posts within the sampled kebeles were visited, the HEWs based there were interviewed and the health post records were reviewed to obtain information on CBDDM implementation strength.

The data collection from households was carried out by health professionals working for regional health bureaus at zonal or woreda levels. They were recruited in consultation with the regional health bureaus. The supervisors were mostly from L10K’s implementing partners. To avoid bias, supervisors and interviewers did not conduct survey work in their own areas. The supervisors were responsible for data collection from the health posts.

Ethical clearance was obtained from the Institutional Review Boards of the respective Regional Health Bureaus and that of the JSI Research & Training Institute, Inc.

### Dependent variables

The maternal and newborn health care practices that were expected to improve due to the intervention were the coverage of four or more antenatal care visits to a health facility (ANC 4+), neonatal tetanus-protected childbirth (defined below), delivery at health facilities, receiving postnatal care within 48 h of childbirth (PNC in 48 h), clean umbilical cord care for home births, thermal care of the newborn, and initiation of breastfeeding within 30 min of childbirth.

If the mother reported receiving at least two tetanus toxoid injections during her lifetime, the last of which had occurred less than 3 years previously; if she received at least three tetanus toxoid injections during her lifetime, the last of which had occurred in the previous 5 years; if she received at least four tetanus toxoid injections during her lifetime, the last of which occurred in the previous 10 years; or if she had received at least five tetanus toxoid injections during her lifetime, her last birth was considered to be protected from neonatal tetanus. Clean cord care was defined as the umbilical cord being cut with a sterile instrument, the cord tied with sterile thread, and either nothing or only chlorhexidine applied to the cut end of the umbilical cord. Thermal care was defined as the newborn being dried and wrapped immediately after birth, delayed bathing of the newborn by 6 hours or more, and maintaining skin-to-skin contact with the baby. The analysis of clean cord care and thermal care was restricted among home deliveries.

### Independent variables

The independent variable of interest for this study was the CBDDM implementation strength. During the follow-up survey, each kebele was assigned a CBDDM implementation strength score based on four items obtained during the health post survey: (a) the proportion of WDA team leaders in the kebele who had a surveillance map of their neighborhood; (b) the proportion of WDA team leaders who had either reported surveillance data to the HEW, or from whom the HEW had collected it, during the last 3 months; (c) whether or not the HEW had updated health post records of surveillance data; and (d) whether the kebele leaders had used surveillance data to monitor the utilization of maternal and newborn health services during the last 3 months. The first three items were measured from WDA activity records maintained by HEWs, while for the fourth item, HEWs were asked if there had been any meeting of kebele leaders (kebele command post meeting) at which maternal and newborn health issues were discussed. If the response was yes, then they were asked whether CBDDM data were used during that meeting. After verifying the responses from meeting minutes, they were recorded.

The first two item scores were probabilities ranging between 0 and 1, while the last two items score were binary responses, where kebeles received a score of 1 for yes and null for no. Each of these was standardized (with mean 0 and variance 1), and then all four were added together to obtain a scale. Cronbach’s alpha, which reflects the internal reliability of the four items in constructing the scale, was 0.75.

The kebeles were ranked according to the score of the scale from the follow-up survey and then divided into three terciles. Due to ties, the three terciles were not equal. There were 68 kebeles in the lowest and middle terciles, and 41 kebeles in the highest tercile. The lowest, middle and highest CBDDM implementation strength score terciles were scored 1, 2, and 3 and named low, medium and high CBDDM implementation strength, respectively. At baseline, the CBDDM implementation strength score was assumed to be zero. Higher scores indicated better CBDDM implementation strength.

The independent variables that were considered as controls for the multivariate analysis were the individual, household, and contextual characteristics of the sample. The individual-level characteristics were age, education, marital status, parity and religion of the respondents; the household-level characteristics were household wealth and distance of the respondents’ household from the nearest health facility; and the contextual characteristics were the HEW-to-population ratio of the kebele, administrative regions, and program stratum. The program stratum was an indicator variable indicating areas in which CBDDM was prototyped prior to the study period.

A single wealth index score was constructed for each household for both survey periods using principal component analysis of household possessions (electricity, watch, radio, television, mobile phone, telephone, refrigerator, table, chair, bed, electric stove, and kerosene lamp) and household characteristics (type of latrine and water source). Households from both the surveys were ranked according to wealth score and then divided into five quintiles [37].

### Statistical analysis

First, we assessed the difference in the characteristics of the respondents between the two survey periods using Pearson’s chi-square statistics adjusted for cluster survey design effects. Then we analyzed the distribution properties of the four items of the CBDDM implementation strength score using box plots. We assessed whether the background characteristics of the respondents were systematically associated with the CBDDM implementation strength score during the follow-up survey using Pearson’s chi-squared statistics adjusted for cluster survey design effects.

We estimated the changes in the outcomes of interest between the two surveys and their 95% confidence intervals using post-estimation procedures following logistic regression models. The survey period was the only predictor in the logit models, and the models accounted for cluster survey design effects, using the Taylor series linearization technique for estimating variances [38].

We estimated the dose-response associations between changes in the CBDDM implementation strength score and the changes in maternal and newborn health care practices using kebele-level random effect models. We used Stata’s ‘*xtlogit*’ command for this purpose [38]. The models accounted for cluster survey design [39], secular change over time, and the individual, household and contextual characteristics of the respondents. We accounted for the secular change of the maternal and newborn health care indicator between the two survey periods by including the survey period as an indicator variable. The CBDDM implementation strength score entered the models as a linear term to identify dose-response association or the trend effect. To assess the contribution of the independent variables to the model, we grouped them into individual, household, and contextual factors. First, we estimated the model with all three groups of independent variables. Then we assessed the statistical significance of each of the three groups one by one using a likelihood ratio test. If a particular group was not statistically significant at *p* < 0.05, we dropped it [40]. We assessed the goodness of fit of the models using global Wald’s statistic and the likelihood ratio test of the kebele-level random effects.

If the linear term for the CBDDM implementation strength score was statistically significant (*p* < 0.05) in the final model, then we tested it for departure from linearity (i.e. not a trend effect). We used the likelihood ratio test to compare the final model with the score as a linear term (restricted model) with the unrestricted model where the score was a categorical variable. If the two models were not statistically significantly different (*p* > 0.05) then we considered the linear term for CBDDM implementation score as adequate, and we concluded that there was a dose-response relationship, or trend effect [41].

Lastly, if we found a trend effect in the CBDDM implementation score, we did a counterfactual analysis to quantify the average treatment effects (ATEs) of CBDDM for maternal and newborn health care practices. We used Stata’s post-estimation ‘margins’ command for the purpose [38]. The regression models were simulated to predict two values for each maternal and newborn health care practice. The first value was the predicted (adjusted) maternal and newborn health care practice when the CBDDM implementation strength score did not change from the baseline value (zero); and the second value was the adjusted maternal and newborn health care practice holding the CBDDM implementation strength score at its mean value during the follow-up survey. The difference between the second and first values estimated the effect of CBDDM on the maternal and newborn health care practice – the ATE.

## Results

We found statistically significant (*p* < 0.05) evidence of change over time between the 2010–11 and 2014–15 surveys for the following background characteristics: education, marital status, parity, household distance from the nearest health facility, and household wealth (Table 1). Compared with the 2010–11 survey, respondents from the 2014–15 survey were more likely to be educated, had fewer children and were wealthier. The increase in wealth between the two surveys was mainly due to the comparatively higher proportion of households with electricity, mobile phones, household furniture, and piped water as the source of drinking water. Contrary to expectations, the respondents from the follow-up survey (2014–15) were more likely to be living further away from health facilities than those in the baseline survey (2010–11). The difference in the marital status of respondents between the survey periods was small.

**Table 1:** Sample characteristics by survey period

Characteristic		2010–11		2014–15		*p*-value
		%	*n*	%	*n*	
Women’s age (years)	15–19	8	168	10	202	0.150
	20–34	77	1629	74	1566	
	35–49	15	327	16	341	
Education	None	73	1559	57	1204	< 0.001
	Primary	16	330	23	487	
	Secondary or higher	11	234	20	417	
Marital status	Single	5	105	4	75	0.034
	In union	95	2019	97	2034	
Number of children	1	21	448	29	609	< 0.001
	2	17	356	18	372	
	3	17	361	14	300	
	4+	45	959	39	827	
Religion	Orthodox	63	1342	62	1302	0.468
	Protestant	19	396	20	431	
	Muslim	17	358	17	355	
	Traditional/other	1	27	1	21	
Distance to any health facility	< 30 min.	64	1365	55	1163	0.002
	30 min - < 1 h	26	545	30	636	
	1 - < 2 h	10	214	15	309	
Wealth quintile	Lowest	28	604	13	256	< 0.001
	Fourth	21	447	18	380	
	Middle	20	426	19	407	
	Second	17	369	23	475	
	Highest	13	279	27	572	
Region	Tigray	13	284	14	286	0.317
	Amhara	38	808	38	791	
	Oromia	28	589	28	583	
	SNNP	21	443	21	449	
Population to HEW ratio	2500 or less	50	1059	47	996	0.407
	2501 – 3500	25	521	25	519	
	3501 – 5000	17	349	15	309	
	> 5000	9	195	14	285	
Program stratum	CBDDM only	76	1606	76	1607	0.427
	Prior program areas	24	518	24	506	
No. of respondents		100	2124	100	2113	

*CBDDM* Community-Based Data for Decision-Making, *HEW* Health Extension Worker, *WDA* Women’s Development Army

**Fig. 2:**
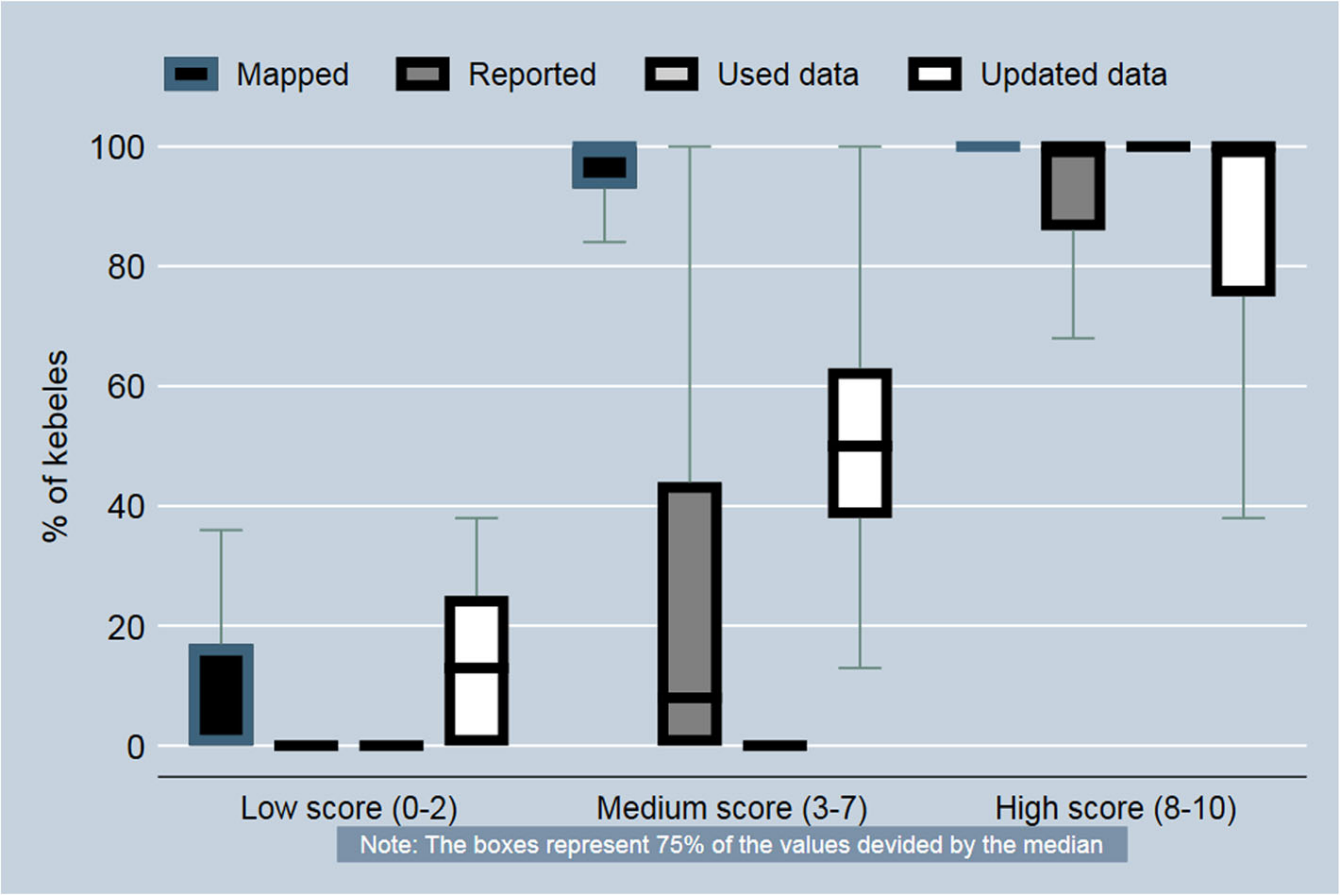
CBDDM activities by CBDDM implementation strength score during 2014–15

The CBDDM implementation strength score during 2010–11 was assumed to be null. During 2014–15, on average 92% of the WDA team leaders had mapped their neighborhood for surveillance, while 48% of them had reported surveillance data to the HEWs. In 63% of the kebeles HEWs were maintaining CBDDM activity registers and 46% of the kebeles were using CBDDM data to monitor access to maternal and newborn health services (Table 2). Figure 2 provides the distribution properties of the CBDDM activity measures according to the CBDDM implementation strength score categories.

**Table 2:** Kebele-level measures of CBDDM implementation strength score and the items, 2014–15

CBDDM Implementation Strength Score Items	CBDDM Implementation Strength			Total
	Low	Medium	High	
% of WDA team leaders on average to have CBDDM map (Mapped)	82	98	99	92
% of WDA team leaders on average who reported CBDDM data in the previous month (Reported)	11	57	95	48
% of kebele command post leaders who used CBDDM data for monitoring maternal and newborn health services in the last 3 months (Used)	9	50	100	46
% of kebeles that have updated CBDDM registers (Updates data)	39	67	97	63
Number of kebeles	68	68	41	177

*CBDDM* Community-Based Data for Decision-Making, *WDA* Women’s Development Army

Table 3 compares sample characteristics of the respondents according to the CBDDM implementation strength score during the 2014–15 survey. Distance of the respondents’ households from the nearest health facility was systematically associated (*p* < 0.05) with CBDDM implementation strength. Respondents in kebeles with relatively high CBDDM implementation strength scores were more likely to live nearer to health facilities. Parity was also associated (*p* < 0.05) with CBDDM implementation strength, but the association did not have any systematic pattern. Although the proportion of respondents in the prior CBDDM program area kebeles appeared to be relatively high in the kebeles with higher CBDDM implementation strength score, this effect was not statistically significant (because the unit of analysis was the kebele, which had a small sample size).

**Table 3:** Sample characteristics by CBDDM implementation strength score at follow-up survey

Characteristic		CBDDM Implementation Strength						*p*-value
		Low		Medium		High		
		%	*N*	%	*N*	%	*N*	
Women’s age (years)	15–19	11	93	9	69	8	40	0.506
	20–34	72	593	76	620	74	356	
	35–49	17	137	15	122	17	82	
Education	None	61	500	57	463	51	244	0.203
	Primary	22	178	23	190	25	121	
	Secondary or higher	18	146	20	159	24	113	
Marital status	Single	3	29	4	28	4	18	0.971
	In union	97	795	97	783	96	460	
Number of children	1	31	257	24	194	33	160	0.032
	2	16	133	21	167	15	74	
	3	13	110	15	119	15	72	
	4+	39	324	41	332	36	173	
Religion	Orthodox	57	465	62	506	70	333	0.179
	Protestant	24	201	15	124	22	107	
	Muslim	18	144	21	174	8	37	
	Traditional/other	2	13	1	7	0	1	
Distance to any health facility	< 30 min.	46	378	59	480	65	309	< 0.001
	30 min - < 1 h	33	275	28	230	28	132	
	1 - < 2 h	21	170	13	102	8	37	
Wealth quintile	Lowest	13	107	14	114	11	55	0.533
	Fourth	18	152	19	156	15	72	
	Middle	21	169	16	133	22	106	
	Second	24	200	22	176	21	99	
	Highest	24	194	29	233	30	146	
Region	Tigray	12	97	14	114	16	74	0.512
	Amhara	31	252	39	314	47	227	
	Oromia	35	287	26	207	19	89	
	SNNP	23	186	22	176	18	88	
Population to HEW ratio	2500 or less	44	365	48	392	50	241	0.533
	2501 – 3500	20	167	21	169	39	184	
	3501 – 5000	13	107	20	162	8	39	
	> 5000	22	183	11	89	3	14	
Program stratum	CBDDM only	81	669	76	618	67	320	0.251
	Prior program areas	19	154	24	193	33	158	
No. of respondents		100	823	100	811	100	478	
No. of communities			68		68		41	

*CBDDM* Community-Based Data for Decision-Making, *HEW* Health Extension Worker

There was no evidence of change (*p* > 0.05) in PNC coverage, while the proportion of women who practiced clean cord care of their newborn among home deliveries declined (*p* < 0.05) between 2010 and 11 and 2014–15 (Table 4). We found some evidence of improvement for all other maternal and newborn health care practices during the observation period (*p* < 0.05).

**Table 4:** Maternal and newborn health care practices during 2010–11 and 2014–15 surveys

Maternal and Newborn Health Care Practice	2010–11	2014–15	Change		
	(%)	(%)	%-points	(95% CI)	*p*-value
ANC 4+	28	52	24	(20, 28)	< 0.001
Neonatal tetanus protected birth	57	63	6	(2, 10)	0.004
Institutional delivery	9	53	43	(39, 48)	< 0.001
PNC in 48 h	10	11	1	(−2, 4)	0.426
Clean cord care among home deliveries	44	38	−6	(−12, 0)	0.036
Thermal care	26	57	30	(26, 35)	< 0.001
Immediate initiation of breastfeeding	53	76	23	(20, 27)	< 0.001

*ANC 4+* received four or more antenatal care visits, *PNC* postnatal care

We used random-effects logit models to assess the linear effects of CBDDM implementation strength on maternal and newborn care practices (Table 5). All the three groups of the respondents’ characteristics were statistically significant (*p* < .05) in all the final models. The kebele-level random effects and the goodness of fit were also statistically significant for all the final models. Assessment of trend effects indicated that the CBDDM implementation strength score had dose-response relationships with several maternal and newborn care practices (Table 5). The odds of neonatal tetanus-protected childbirth, institutional deliveries, clean cord care and thermal care of the newborn, and initiation of breastfeeding immediately after childbirth increased respectively by 23%, 47%, 21%, 29% and 27% when we compared kebeles with low CBDDM implementation strength (at follow-up) with kebeles with no CBDDM implementation strength (at baseline); or when we compared kebeles with medium CBDDM implementation strength with kebeles with low CBDDM implementation strength; or when we compared kebeles with high CBDDM implementation strength with kebeles with medium CBDDM implementation strength. However, we found no evidence that CBDDM implementation strength was associated with utilization of four or more antenatal care services and early postnatal care, which we defined as a visit within 2 days of delivery.

**Table 5:** Linear effects of CBDDM implementation strength score and test of trend

Maternal and Newborn Health Care Practice	Linear Effects of CBDDM			Test for Trend	
	OR	(95% CI)	*p*-value	*p*-value^a^	Dose-Response Relationship
ANC 4+	1.12	(0.99, 1.26)	0.067	ND	No
Neonatal tetanus protected birth	1.23	(1.09, 1.38)	< 0.001	0.056	Yes
Institutional delivery	1.47	(1.25, 1.73)	< 0.001	0.748	Yes
PNC in 48 h	1.07	(0.91, 1.27)	0.418	ND	No
Clean cord care among home deliveries	1.21	(1.01, 1.44)	0.039	0.827	Yes
Thermal care	1.29	(1.08, 1.55)	0.006	0.136	Yes
Immediate initiation of breastfeeding	1.27	(1.10, 1.46)	0.001	0.596	Yes

*CBDDM* Community-Based Data for Decision-Making, *OR* odds ratio, *ND* not done, *ANC 4+* received four or more antenatal care visits, *PNC* postnatal care. ^a^Null hypothesis: Not a trend effect

The ATEs of CBDDM on maternal and newborn care practices ranged between 8 and 15 percentage-points (Table 6). For example, 15 percentage-points of the institutional delivery rate at follow-up were attributable to the CBDDM strategy.

**Table 6:** Average treatment effects (ATEs) of CBDDM from simulation of models that concluded dose-response relationships

Maternal and Newborn Health Care Practice	ATE of CBDDM		
	%-points	(95% CI)	*p*-value
Neonatal tetanus protected birth	9	(4, 14)	0.001
Institutional delivery	15	(9, 20)	< 0.001
Clean cord care among home deliveries	7	(1, 14)	0.030
Thermal care	10	(3, 17)	0.003
Immediate initiation of breastfeeding	8	(3, 13)	0.002

*ATE* average treatment effect, *CBDDM* Community-Based Data for Decision-Making

## Discussion

We sought to assess the effectiveness of a community-based health information system, designed for a low-literacy community health volunteer program and implemented at scale, to improve maternal and newborn care behaviors and practices. We found strong evidence of a dose-response relationship between the implementation strength of CBDDM and better care practices, indicating that the strategy was effective. The findings were consistent with an earlier analysis of the effects of CBDDM on the utilization of maternal and newborn health care services, using program monitoring data and service statistics [42]. Engaging communities to collect and use data to plan and improve the uptake of maternal and newborn care services has been effective in other developing country settings [18, 23, 24].

The HEWs were mainly responsible for supporting the WDA to implement the CBDDM strategy. They used the strategy to provide targeted maternal and newborn health services and to maintain the Community Health Information System. Thus, the observed effects of CBDDM on maternal newborn care practices may partly reflect the efforts of HEWs, including the influence of the Community Health Information System. The effects of CBDDM are likely to be mediated by HEWs, including through their use of the Community Health Information System. Nonetheless, we concluded that the CBDDM strategy most likely improved the efficiency of the Health Extension Program.

With only 3.2 skilled health workers (doctors, nurses, and midwives) per 10,000 people, in 2012 [43], Ethiopia was far from the World Health Organization’s recommended minimum of 23 skilled health workers per 10,000 people required to provide the health coverage needed to achieve the health-related Millennium Development Goals (MDGs) [44]. As it was committed to reach the health-related MDGs, the Federal Ministry of Health addressed the shortfall of skilled health workers by establishing the Health Extension Program in 2003 and task shifting some of the basic maternal, newborn and child health services from skilled health workers to HEWs, with a more basic level of training [31, 32, 45]. The Health Extension Program contributed towards achieving the MDG target for child survival [46]. Thus, the Health Extension Program is expected to play a key role in achieving the health-related targets of the Sustainable Development Goals set for 2030. Realizing that CBDDM is a useful tool for the WDA to carry out its responsibilities, mainly facilitating the uptake of high-impact interventions by underserved households, the Health Extension Program has adopted it within its national WDA program to improve its efficiency.

The recent and impressive increase in institutional deliveries in Ethiopia —from 10% in 2011 to 28% in 2016 [47]— is likely due to national government strategies, including mobilizing communities to encourage pregnant mothers to give birth in health facilities, creating effective supportive and referral linkages between communities and health facilities, staffing health centers with midwives to ensure the continuous availability of basic emergency obstetric care services and the provision of ambulances to districts to mitigate transportation barriers [48]. The current study indicates that the CBDDM strategy augmented this national effort.

The practice of clean cord care among home deliveries declined during the observation period. During 2010–11 almost all women were delivering at home. As more and more women give birth at health facilities, over time, it is likely that those who deliver at home will be less aware of the best maternal and newborn health care behaviors and practices. However, in kebeles where the CBDDM implementation strength was relatively low, the decline in clean cord care was also relatively low.

Interestingly, we found no evidence that CBDDM affected early postnatal care. Further investigations will be required to identify the barriers and to address them.

There are several limitations to this study. First, the household selection for data collection can be criticized because the interviewers may have avoided hard-to-reach areas and non-responders were not revisited [36]. The increase in the average distance of the respondents’ household from the nearest health facility between the two survey periods most likely indicates that the interviewers during the 2014–15 survey made greater efforts to interview households from hard-to-reach areas than those during the 2010–11 survey. The possible bias that the change in the households’ distance to the nearest health facility could introduce was minimized by controlling for distance to any health facility in our analysis and by using multi-level analysis. Second, the outcomes considered are associated with the most recent birth among women with surviving children aged 0 to 11 months old, excluding women whose children died before the women had the opportunity for an interview. It is likely that such cases would have relatively poor practices; their exclusion would, therefore, result in overestimating care practices. Nevertheless, there are few such women and also, since the sampling method was consistent between the surveys, the sampling strategy to interview women with living children does not affect the internal validity of this study. Third, the CBDDM effects could be biased due to unmeasured confounders. The respondents living in kebeles with relatively high CBDDM implementation strength were nearer to health facilities. Although we controlled for distance of the respondents’ household from the nearest health facility, there could be other unmeasured confounders. For example, the effects to CBDDM could be overestimated if the CBDDM implementation strength was higher in kebeles where there have been improvements in other unmeasured developmental factors that also influenced maternal and newborn care practices. Lastly, there could be reverse causality; kebeles with better household practices selecting more motivated WDA team leaders who implemented the CBDDM strategy better.

## Conclusion

The CBDDM strategy demonstrated that data can be obtained and used by community members who have no or little education and used at the community level to support Ethiopia’s Health Extension Program —thus underscoring the importance of a local information system to engage communities effectively to improve the performance of health systems. Potentially, the strategy could be adapted to community-based maternal and newborn health care systems elsewhere that have similar settings. Nonetheless, further research will be required to understand why the strategy fell short in improving the coverage of early postnatal care.

## Abbreviations

ANC 4+: Four or more antenatal care visits at a health facility
ATE: Average treatment effect
CBDDM: Community-Based Data for Decision-Making
CI: Confidence interval
HEW: Health Extension Worker
L10K: The Last Ten Kilometers
MDG: Millennium Development Goal
PNC: Postnatal care
SDG: Sustainable Development Goal
SNNP: Southern Nations, Nationalities and Peoples
WDA: Women’s Development Army

## References

[1] Tunçalp Ӧ, Were WM, MacLennan C, Oladapo OT, Gülmezoglu AM, Bahl R (2015). Quality of care for pregnant women and newborns - the WHO vision. BJOG.

[2] Pathé S, Habib B, Joses S, Kirigia M, Nyoni J, Bessaoud K (2010). The Ouagadougou declaration on primary health care and health systems in Africa: achieving better health for Africa in the new millennium. Afr Health Monit.

[3] World Health Organization (WHO) (2011). Rio political declaration on social determinants of health.

[4] Liu A, Sullivan S, Khan M, Sachs S, Singh P (2011). Community health workers in global health: scale and scalability. Mt Sinai J Med.

[5] Singh P, Sachs JD (2013). 1 million community health workers in sub-Saharan Africa by 2015. Lancet.

[6] Campbell C, Jovchelovitch S (2000). Health, community and development: towards social psychology of participation. J Community Appl Psychol.

[7] Jewkes R, Murkott A (1998). Community representatives: representing the “community”?. Soc Sci Med.

[8] Marston C, Renedo A, McGowan CR, Portela A (2013). Effects of community participation on improving uptake of skilled care for maternal and newborn health: a systematic review. PLoS One.

[9] Global Fund (2014). Community systems strengthening framework.

[10] Marsh D, Lippeveld T (2000). Population-based health information systems. Design and implementation of health information systems.

[11] Azim T, Tilahun B, Mullen S (2018). Use of community health data for shared accountability: guidance.

[12] de la Torre C, Unfried K (2014). Monitoring and evaluation at the community level: a strategic review of MEASURE evaluation, phase III accomplishments and contributions.

[13] Rosales A (2003). A community based health information system in rural Honduras: CRS/COCEPRADII experience.

[14] Chewicha K, Azim T (2013). Community health information system for family centered health care: scale-up in southern nations nationalities and People’s region. Ethiop Minist Heal Q Heal Bull.

[15] Damtew ZA, Moges AS (2013). From multiple register to family folder: the transition of data collection and reporting tools for health extension Workers in Ethiopia. J Health Inform Dev Ctries.

[16] MEASURE Evaluation. Community-based health information systems in the global context: A review of the literature. Chapel Hill: MEASURE Evaluation; 2016. Report No.: WP-16-161. https://www.measureevaluation.org/resources/publications/wp-16-161. Accessed 15 Apr 2018.

[17] Odhiambo-otieno GW, Management H, Health P (2005). Implementing a community-based health management information system in Bungoma district, Kenya. Heal Policy Dev.

[18] Sabitu K, Iliyasu Z, Hassan SS, Mande AT (2004). Evaluation of a community level nutrition information system for action in a rural community of Zaria, Northern Nigeria. Ann Afr Med.

[19] Jeremie N, Kaseje D, Olayo R, Akinyi C (2014). Utilization of community-based health information systems in decision making and health action in Nyalenda, Kisumu County, Kenya. Univers J Med Sci.

[20] MEASURE Evaluation (2014). Case study series: community-based information systems: Tanzania.

[21] MEASURE Evaluation (2014). Case study series: community-based information systems: Kenya.

[22] MEASURE Evaluation (2014). Case study series: community-based information systems: Zambia.

[23] Kaseje D, Olayo R, Musita C, Oindo C, Wafula C, Muga R (2010). Evidence-based dialogue with communities for district health systems’ performance improvement. Glob Public Health.

[24] Björkman M, Svensson J (2009). Power to the people: evidence from a randomized field experiment on community-based monitoring in Uganda. Q J Econ.

[25] Akinyi C, Nzanzu J, Kaseje D (2015). Effectiveness of community health workers in promotion of maternal health services in Butere district, rural western Kenya. Univers J Med Sci.

[26] Editorial (2010). Evaluation: the top priority for global health. Lancet.

[27] Damtew ZA, Karim AM, Willey B, Tesfaye C, Fesseha Zemichael N, Yeshanew B, et al. Correlates of the Women’s Development Army strategy implementation strength with household reproductive, maternal, newborn and child healthcare practices: a cross-sectional study in four regions of Ethiopia. BMC Pregnancy Childbirth. 2018;18(Suppl 1) 10.1186/s12884-018-1975-y. 10.1186/s12884-018-1975-y PMC6157249 30255789

[28] Wereta T, Betemariam W, Karim AM, Fesseha Zemichael N, Dagnew S, Wanboru A, et al. Effects of a participatory community quality improvement strategy on improving household and provider health care behaviors and practices: a propensity score analysis. BMC Pregnancy Childbirth. 2018;18(Suppl 1) 10.1186/s12884-018-1977-9. 10.1186/s12884-018-1977-9 PMC6157250 30255783

[29] Emaway Altaye D, Karim AM, Betemariam W, Fesseha Zemichael N, Shigute T, Scheelbeek P. Effects of family conversation on health care practices in Ethiopia: a propensity score matched analysis. BMC Pregnancy Childbirth. 2018; 18(Suppl 1) 10.1186/s12884-018-1978-8. 10.1186/s12884-018-1978-8 PMC6157286 30255781

[30] Federal Ministry of Health, Ethiopia (2015). Health Sector Transformation Plan.

[31] Wakabi W (2008). Extension workers drive Ethiopia’s primary health care. Lancet.

[32] Admasu K, Balcha T, Getahun H (2016). Model villages: a platform for community-based primary health care. Lancet.

[33] Karim AM, Admassu K, Schellenberg J, Alemu H, Getachew N, Ameha A (2013). Effect of Ethiopia’s health extension program on maternal and newborn health care practices in 101 rural districts: a dose-response study. PLoS One.

[34] Federal Ministry of Health, Ethiopia (2016). The Health Development Army: Its origins, development and current status, The Health Documentation Initiative.

[35] Darmstadt GL, Marchant T, Claeson M, Brown W, Morris S, Donnay F (2013). A strategy for reducing maternal and newborn deaths by 2015 and beyond. BMC Pregnancy Childbirth.

[36] Lemeshow S, Robinson D (1985). Surveys to measure programme coverage and impact: a review of the methodology used by the expanded programme on immunization. World Heal Stat Q.

[37] Filmer D, Pritchett L (2001). Estimating wealth effects without expenditure data– or tears: an application to educational enrollment in states of India. Demography.

[38] StataCorp (2015). Stata base reference manual, Release 14.

[39] Angeles G, Guilkey DK, Mroz T (2005). The impact of community-level variables on individual-level outcomes: theoretical results and applications. Sociol Methods Res.

[40] Hosmer D, Lemeshow S (1989). Applied logistic regression.

[41] Clayton D, Hill M (1993). Statistical models for epidemiology.

[42] Karim A (2015). Effectiveness evaluation of a large-scale community-based program: lessons from Ethiopia. Ann Glob Heal.

[43] Feysia B, Herbst CH, Lemma W, Soucat A, Zhao F, Kedir N (2012). The health workforce in Ethiopia: addressing the remaining challenges.

[44] World Health Organization (2006). Working together for health: the world health report 2006.

[45] Bhutta Z, Lassi Z, Pariyo G, Huicho L. Global experience of community health workers for delivery of health related millennium development goals: a systematic review, country case studies, and recommendations for integration into national health systems. Community Dent Health. 2010; http://www.who.int/workforcealliance/knowledge/publications/alliance/Global_CHW_web.pdf. Accessed 17 Oct 2017

[46] Ethiopia Public Health Institute, Federal Ministry of Health, Countdown to 2015, UNICEF (2015). Countdown to a healthier Ethiopia: building on successes to accelerate newborn survival.

[47] Central Statistical Agency, Ethiopia (2016). Ethiopia Demographic and Health Survey 2016 Key indicators.

[48] Admasu K, Balcha T, Ghebreyesus TA (2016). Pro–poor pathway towards universal health coverage: lessons from Ethiopia. J Glob Health.

